# Regulation of miR394 in Response to *Fusarium oxysporum* f. sp. *cepae* (FOC) Infection in Garlic (*Allium sativum* L)

**DOI:** 10.3389/fpls.2016.00258

**Published:** 2016-03-04

**Authors:** Subodh K. Chand, Satyabrata Nanda, Raj K. Joshi

**Affiliations:** Functional Genomics Laboratory, Centre of Biotechnology, Siksha O Anusandhan UniversityBhubaneswar, India

**Keywords:** *Allium sativum*, *Fusarium oxysporum* f. sp. *cepae*, miR394, jasmonic acid, CYP450, F-box, RLM-5′ RACE

## Abstract

MicroRNAs (miRNAs) are a class of post-transcriptional regulators that negatively regulate gene expression through target mRNA cleavage or translational inhibition and play important roles in plant development and stress response. In the present study, six conserved miRNAs from garlic (*Allium sativum* L.) were analyzed to identify differentially expressed miRNAs in response to *Fusarium oxysporum* f. sp. *cepae* (FOC) infection. Stem-loop RT-PCR revealed that miR394 is significantly induced in garlic seedlings post-treatment with FOC for 72 h. The induction of miR394 expression during FOC infection was restricted to the basal stem plate tissue, the primary site of infection. Garlic miR394 was also upregulated by exogenous application of jasmonic acid. Two putative targets of miR394 encoding F-box domain and cytochrome P450 (CYP450) family proteins were predicted and verified using 5′ RLM-RACE (RNA ligase mediated rapid amplification of cDNA ends) assay. Quantitative RT-PCR showed that the transcript levels of the predicted targets were significantly reduced in garlic plants exposed to FOC. When garlic cultivars with variable sensitivity to FOC were exposed to the pathogen, an upregulation of miR394 and down regulation of the targets were observed in both varieties. However, the expression pattern was delayed in the resistant genotypes. These results suggest that miR394 functions in negative modulation of FOC resistance and the difference in timing and levels of expression in variable genotypes could be examined as markers for selection of FOC resistant garlic cultivars.

## Introduction

Fusarium basal rot (FBR) of garlic (*Allium sativum*) is a notorious disease caused by a soil borne fungal pathogen, *Fusarium oxysporum* f. sp. *cepae* (FOC; Matou et al., 1986). The pathogen colonizes on the root surface and enters into garlic through the wounded area on the basal stem plate. Under a conducive environment, the pathogen develops very quickly and the plants shows discoloration of basal plate tissues, root abscission, leaf necrosis and eventually die soon (Cramer, 2000). Besides, FBR provides a mode of entry for secondary pathogens to infect the bulb scales. This fungus is singly responsible for 60% yield losses at both pre- and post-harvest stage, and therefore is regarded as a major threat to garlic production (Cramer, 2000). To date, there is no perfect strategy for control or cure of this disease because the mechanism underlining FBR incidence is not clear yet.

The molecular and biochemical mechanisms governing the interaction between *F. oxysporum* and *Arabidopsis thaliana* have been extensively studied in the recent times. Evidences indicate that *F. oxysporum* produces bioactive jasmonates which promote host senescence (Cole et al., 2014). On the other hand, the JA receptor mutant *coi1* exhibit high resistance to *F. oxysporum* (Thatcher et al., 2009). This suggests that JA signaling have both positive and negative effects toward *F. oxysporum* in *A. thaliana*. Besides, other phytohormones also regulate the host response to *F. oxysporum*. While ethylene and abscisic acid (ABA) promotes susceptibility to the pathogen, salicylic acid (SA) promotes resistance by activating the SA mediated signaling in the biotrophic phase of *F. oxysporum* infection. Analysis of the defense transcriptome responsive to *F. oxysporum* infection in *A. thaliana* showed that many novel disease responsive genes including wall associated kinases (WAKs), RPM1-interacting protein4 (RIN4), and Cytochrome P450 (CYP450) genes were induced (Zhu et al., 2013). Besides, many non-coding RNAs such as miR159, miR319, and transcriptionally active region 224 (TAR224) were demonstrated to be related to disease development (Zhu et al., 2013, 2014). A recent work on transcriptional changes in *A. thaliana* post-infection with *F. oxysporum* revealed that the pathogen triggers tissue-specific regulation of host genes (Lyons et al., 2015). The same group reported that the genetic regulators of auxins and ABA signaling are strongly regulated in the root tissue, while the genes associated with jasmonate biosynthesis and signaling were expressed through the plant. However, the actual genetic networks associated with FOC-garlic interaction is still poorly understood mainly due to lack of more comprehensive genome sequence knowledge.

Plants have evolved a complex network of cellular, physiological and molecular responses to counter act against multiple stresses including environmental cues and invasion by phytopathogens (Rejeb et al., 2014). Biotic stresses such as invasion of bacteria, viruses, fungi, and insect predators regulate the expression of thousands of genes in plants at both the transcriptional and post-transcriptional levels. In recent years, a group of negative regulators called microRNAs (miRNAs), have been identified as prominent determinants of post-transcriptional gene regulation and exhibit an important function in regulation of plant growth and stress responses (Sunkar et al., 2007; Leung and Sharp, 2010). miRNAs are 20–24 nucleotide (nt) small regulatory RNAs that recognize mRNA targets through sequence complementarity and down regulate the expression of the target genes by cleavage or repression of translation (Jones-Rhoades et al., 2006; Shukla et al., 2008). Increasing evidence indicates that miRNA have greatly expanded roles in many plant biological and metabolic processes including regulation of plant development, signal transduction, and response to abiotic stresses and pathogen invasions (Shukla et al., 2008; Chuck et al., 2009; Liu and Chen, 2009; Yang et al., 2015).

Recent studies have shown that a multitude of miRNAs play critical roles in plant-microbe interaction toward defense responses (Navarro et al., 2006; Padmanabhan et al., 2009; Yang and Huang, 2014). Plant miRNAs and the mediated RNA interference (RNAi) pathaway components are critical to plant immunity against bacteria, viruses and fungi (Weiberg et al., 2014). The first indication of such role came from *A. thaliana* miR393 which contributed to pathogen associated molecular pattern triggered immunity (PTI; Navarro et al., 2006). miR393 is induced by flg22, a pathogen associated molecular pattern (PAMP) and targets the auxin receptor TIR1 and its homologs thereby cleaving them and suppressing the auxin signaling pathway. In addition to this, several miRNAs including miR160, mi168, miR398, and miR773 regulates flg22 induced callose deposition as part of the PTI response (Li et al., 2010). Negative regulation of an F-box gene by miR393 in *Zea mays* plays an important role in defense against *Rhizoctonia solani* infection (Luo et al., 2014). Plant miRNAs responsive to fungal infection has also been identified in many plant species. Small RNA profiling of *Magnaporthe oryza* challenged resistant and susceptible cultivars of rice revealed that miR156, miR160, miR169, miR164 are induced whereas miR394, and miR396 were down regulated upon infection in resistant cultivars but not in susceptible one (Li et al., 2014). In addition, miR169, miR172, and miR398 were induced in both resistant and susceptible cultivars suggesting their dogmatic role in basal responses (Li et al., 2014). Furthermore, overexpression of miR160 in a susceptible rice cultivar led to enhanced disease resistance toward *M. oryza* suggesting their critical involvement in defense response against fungal pathogen (Li et al., 2014). Besides, several studies on oil seed rape, wheat and tomato have demonstrated the possible role of conserved and novel miRNA-mediated gene silencing in plant defense against multiple fungal phytopathogens (Xin et al., 2010; Campo et al., 2013; Shen et al., 2014; Jin and Wu, 2015). Recently miRNAs have been suggested as chief regulators of nucleotide binding site leucine rich repeat (NBS-LRR) defense gene family through production of trans acting small interfering RNAs (ta-siRNAs; Zhai et al., 2011). Genome wide sequencing has led to the identification of microRNA targeted NBS-LRR genes in a number of plant species including grapevine, sugarcane, loblolly pine, and eggplant (Yang and Huang, 2014). More recently, Yang et al. (2015) reported that the over expression of miR482e targeting NBS-LRR genes caused high susceptibility to *Verticillium dahlia* infection in potato. Emerging evidences also indicate that immune-regulated miRNAs that are differentially regulated upon pathogen attack are translocated into interactive organisms and induce cross-kingdom RNA interference (RNAi; Knip et al., 2014). While the host induced RNAi triggers the silencing of pathogen genes in a process referred to as HIGS, pathogen secreted small RNAs also mimics host miRNAs and suppresses plant immunity (Weiberg et al., 2015; Weiberg and Jin, 2015). For example, three *Botrytis cinerea* small RNAs (Bc-siRNAs) impersonate plant miRNAs and suppresses *Arabidopsis* and tomato defense responsive genes *in vivo* (Weiberg et al., 2013). All these reports clearly suggest that sRNAs and especially miRNAs play critical role in fine tuning the interaction between plant and the pathogen toward reprogramming of immune responses.

In one of our recent study, *de novo* analysis led to the identification of 151 known and 11 novel miRNAs responsive to FOC infection in garlic (unpublished). However, the regulatory mechanism of these miRNAs and their temporal/spatial expression pattern in response to FOC infection still remain unknown. In the present work, we studied the involvement of a selected set of defense responsive miRNAs and their targets in the resistance to FOC infection in garlic. The results reported here indicated the regulation of miR394 and its targets in garlic post-inoculation with FOC. Further, as the Jasmonic acid (JA) signaling has a significant role in triggering defense response against necrotrophic infection (Antico et al., 2012), we analyzed the involvement of JA in the regulation of miR394 during FOC infection. Furthermore, we also evaluated the differences in the timing and intensity of the miR394 regulation between garlic cultivars that are resistant and susceptible to FBR.

## Materials and methods

### Plant material, stress treatment, and RNA isolation

Garlic cloves from *A. sativum* cv. Yamuna Safed 4 (YS4) were treated with 7% hypochlorite solution for 30 min followed by three washes with sterilized water and sowed in pots with autoclaved soil. The cloves were grown in growth chamber with 12 h of light and dark period at 25°C and 60–65% relative humidity. Ten day old seedlings, free of micro-organisms were used for FOC (strain FOC-CBT3) inoculation according to the procedures previously described (Rout et al., 2015). The garlic pots were added with 40 ml of conidial suspension and incubated in a growth chamber at 26/27°C with 12 h photoperiod. Control plants were mock inoculated with sterile distilled water. Control and treated samples were harvested at 0, 6, 12, 24, 48, and 72 h after treatment. A set of five plants was collected for each time point of the experiment and pooled together for the analysis. In a second experiment of FOC infection, a set of garlic YS4 seedlings were inoculated with FOC for 0, 24, 48, and 72 h and roots, stems, and leaves were collected separately. For experiment with MeJA, garlic seedlings were treated with 100 μM MeJA for a period of 0, 6, 12, 24, 48, and 72 h. Plants grown in medium without MeJa were used as controls for each time points. The experiment was done in duplicate with a set of three plants collected for each time point.

Two *A. sativum* accessions CBT-As153 and CBT-As21 with variable sensitivities to FOC infection were used to compare the expression of miRNAs and their targets in garlic. CBT-As153 had shown high level of resistance to three virulent FOC isolates and is major source of disease resistance genes (Rout et al., 2014, 2015). In contrast, CBT-As11 has been reported as a highly susceptible line (Rout et al., 2015). Seedlings were grown in sterilized pots and transplanted into fresh pots with same soil substrate 40 days after sowing in the greenhouse. After 10 days, the plants were expose to FOC infection and plants with treated and control shoots were collected after 0, 24, 48, and 72 h of treatment. Plant samples were immediately frozen in liquid nitrogen and grounded into fine powder. Total RNA was extracted from the frozen samples using TRI reagent (Sigma Aldrich, USA) and treated with DNAse I (Promega, Madison, WI) as per manufacturer's instructions. Total RNA quantity and purity was assayed using a NanoDrop ND-1000 spectophotometer (Thermo Scientific, Waltham, USA) and the RNA samples with 260/280 nm ratio between 2.0 and 2.1 were used for further analysis.

### Validation of experimental stress

Pathogenesis related protein (*AsPR5*) and nucleotide binding site-leucine rich repeat (NBS-LRR) protein (*AsR1*) has been reported to be induced by FOC infection in garlic (Rout et al., 2014, 2016). In the present study, the expression analysis of these two genes was evaluated through qRT-PCR to verify the efficiency of FOC infection in garlic. Treatment with JA was also verified with both the genes. Gene specific primers for *AsPR5* and *AsR1* were (forward: CATGCAGACTCACTGTCCTTGA; reverse: GAGTGCGACTGAAAGCGCACCA) and (forward: GCGTGAGCCAGAAATTTAGC; reverse: ATCGGTTTCCCACACATCAT), respectively. The first strand cDNA was synthesized by reverse transcribing 2 μg RNA using SuperScript II reverse transcriptase (Life Technologies). qRT-PCR was set in a total volume of 10 μL reaction mixture containing 1 μL of reverse transcribed cDNA, 2 μL each of the gene specific primer pairs (5 μM), 5 μL FASTSYBR Green PCR Reagent mix (Life Technologies, Burlington, ON, Canada) and 2 μL of nuclease free water. All reactions were performed in triplicate using a StepOne plus real time PCR system. Constitutively expressed housekeeping gene *actin*1 from *A. sativum* was used to normalize the concentration of qRT-PCR products. The relative expression was calculated using the control for the same time point according to comparative 2^−ΔΔCt^ method (Livak and Schmittgen, 2001).

### Expression analysis of mature miRNAs

Stem-loop reverse transcription PCR was performed to determine the expression profiles of 6 garlic mature miRNAs (miR156, miR159, miR169, miR319, miR394, and miR482; Table S1). The six miRNAs selected in this study have been previously demonstrated to have regulatory roles in plant-microbe interactions (Balmer and Mauch-Mani, 2013; Zhu et al., 2013). These miRNAs were identified from the garlic cultivar YS4 through a *de novo* genome wide small RNA sequencing (unpublished). The stem-loop RT primers and miRNA specific forward primers (Table S2) were designed by following the method described by Varkonyi-Gasic et al. (2007). First strand cDNA synthesis was carried out with 2 μg RNA, 1 μL 15 mer Oligo-dT primer (500 ng/ μL), 2 μL of 10 mM dNTP, 2 μL of 10X RT buffer, 200 units of *MMuLV* reverse transcriptase (Promega, Madison, WI), and 1 μL of miRNA specific stem-loop primer (1 μM). The first strand cDNA was used as template to perform qRT-PCR with SYBR Green PCR master mix (Life Technologies, Burlington, ON, Canada). A 10 μL reaction mixture consisting of 5 μL FASTSYBR Green solution, 1 μL of first strand cDNA, 2 μL of nuclease free water, 1 μL of miRNA specific forward primer and 1 μL of universal reverse primer (5′-GTGCAGGGTCCGAGGT-3′). The relative expression of the mature miRNAs was analyzed according to 2^−ΔΔCt^ method as described previously.

### North blot analysis

Total RNA from the shoots of YS4 seedlings isolated during the second experiment was separated on a denaturing 15% poly acrylamide gel. Then the RNA was transferred to a Ambion Bright Star Plus positively charged nylon membrane (Life Technologies, Carlsbad, USA) by capillary method and fixed by ultra violet cross linking. Biotinylated probes were designed against miR394 using the MAXI script labeling kit (Life Technologies, Carlsbad, USA). Blots were hybridized using the Northern Max kit which includes the ULTRA hyb hybridization buffer (Life Technologies, Carlsbad, USA). Biotinylated probe and target hybridization was detected using the Bright Star Bio detect Non isotopic detection kit (Life Technologies, Carlsbad, USA) according to the manufacturer's instructions.

### Identification and analysis of miRNA targets

Putative target genes for miR394 were identified using the PsRNATarget server (Dai and Zhao, 2011). A pair wise homolog search was carried out by subjecting mature miRNAs as query against manually curated *de novo* assembled garlic transcriptome sequences. No more than 3 mismatches between the sRNA and target were allowed. Other parameter for identification of miRNA targets includes length of complementary scoring: 20; maximum energy to unpair the target site (UPE) ≥20; flanking length around target site for target accessibility analysis: 17 bp upstream and 13 bp downstream; range of central mismatch leading to translational inhibition: 9–11 nt. Expression analysis of the putative targets was evaluated by qRT-PCR using gene specific primers (Table S3).

### Validation of miRNA target through RLM-5′ RACE assay

RNA ligase mediated 5′ rapid amplification of cDNA ends (RLM-5′ RACE) was carried out for mapping of cleaved sites and validation of predicted targets using the Gene Racer kit (Life Technologies). Two micrograms of the total RNA was ligated to a 5′ RACE RNA oligo adaptor sequence. First strand cDNA synthesis was carried out using random hexamer primer. PCR amplification of the cDNA fragment with the cleaved site of the target was performed using a 5′ RACE primer and a gene specific outer primer (Table S4). A touch down PCR was performed in a Veriti gradient thermal cycler (Applied Biosystems) with temperature conditions as follows: 94°C for 2 min, followed by five cycles at 94°C for 30 s and 72°C for 90 s, five cycles at 94°C for 30 s and 72°C for 120 s, and 25 cycles at 94°C for 30 s, 65°C for 30 s and 72°C for 90 s and final extension at 72°C for 10 min. A second nested PCR was performed using a nested 5′ RACE primer, gene specific nested primer and 2 μL of the initial PCR reaction as the template. The PCR conditions were 2 min at 94°C followed by 25 cycles of 30 s at 94°C, 30 s at 65°C, 2 min at 72°C and final extension of 10 min at 72°C. The RACE amplified products were separated on a 1.2% agarose gel, purified and cloned into the pGEM-T easy vector (Promega, Manheim, Germany) and sequenced.

### Statistical analysis

The qRT-PCR results were analyzed by using the StepOne software v 2.2.1. The statistical significances of the results were analyzed with two-way analysis of variance (ANOVA) and multiple comparisons were performed using uncorrected Fischer's least significant difference (LSD) test. The mean values were considered to be significantly different at *p* < 0.05.

## Results

### Expression of miRNAs in FOC infected garlic plants

**Figure 1:**
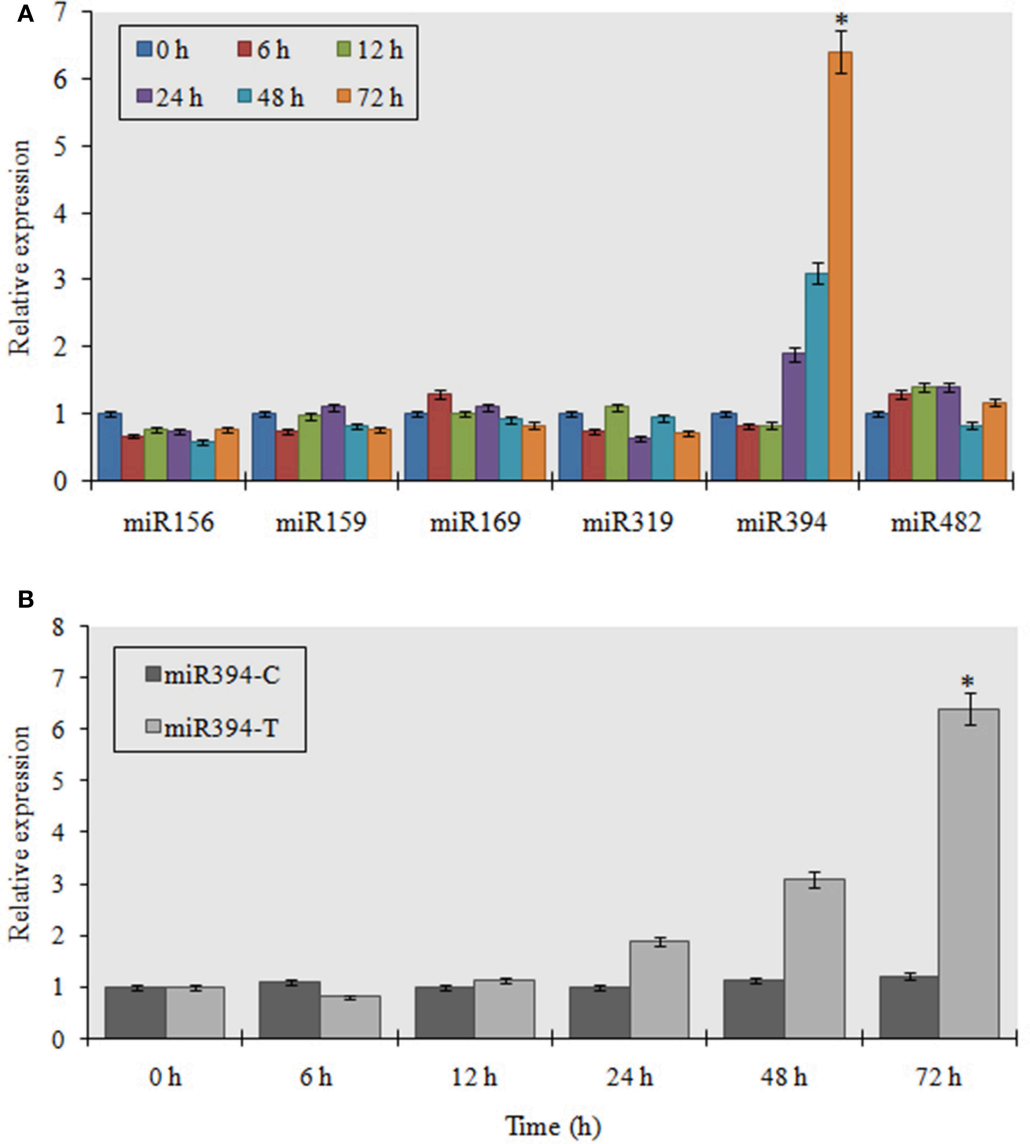
**Analysis of garlic miRNA expression in response to FOC. (A)** Entire plants of garlic YS4 grown *in vitro were* subjected to FOC infection. A set of five plants was collected after 0, 6, 12, 24, 48, and 72 h of treatment. Every point of the experiment had its own control. Analysis of the expression of 6 stress responsive mature miRNAs was performed by pulsed real-time PCR (RT-PCR). The level of transcript is represented as relative expression of each miRNA with respect to its respective control. C, control; T, inoculated with FOC. The housekeeping gene used as control was garlic *Actin*. Error bars shows standard deviations for three independent experiments in real-time PCR. ^*^Indicates the significant difference (at *P* < 0.05) between infected and mock samples identified through two-way ANOVA test. **(B)** Temporal expression pattern of miR394 in response to FOC using quantitative real time PCR.

To verify the expression of stress responsive miRNAs, garlic plants were exposed to FOC for various time periods. The symptoms of FOC infection are hardly visible in the small seedlings (Figures S1A,B). However, the garlic clove show spongy, sunken, yellow-brown rotten lesions gradually rising from the basal plate region (Figure S1C). In the storage bulb, the symptoms appears as reddish or reddish purple discoloration on stem plate and bulb within 72 hpi gradually resulting in deep crack in the cloves followed by decaying of the bulb tissue (Figure S1D). At first, we analyzed the qRT-PCR expression of two marker genes—*AsPR5* and *AsR1* in infected and control plants to validate FOC infection in garlic. The results showed that *AsPR5* was induced 3.8-, 2.4-, 4.1-, and 2.7-fold at 12, 24, 48, and 72 h, respectively. Similarly, *AsR1* was induced 3-, 5.43-, and 4.32-fold at 24, 48, and 72 h, respectively, (Figure S2). This indicates that the FOC treatment was effective in garlic plants. This was followed by expression analysis of six miRNAs by stem-loop RT-PCR. These miRNAs have been implicated in response to fungal phytopathogen (Balmer and Mauch-Mani, 2013; Yang and Huang, 2014). Relative expression analysis of the miRNAs revealed that miR394 was significantly induced while other miRNAs showed small differences in expression as compared to control plants (Figure 1A). As shown in Figure 1B, transcript levels of miR394 notably increased from 24 to 72 h after inoculation as compared to the mock-inoculated control. miR394 was induced 1.8-, 3.1-, and 6.4-fold at 24, 48, and 72 h, respectively. Furthermore, the result of the semi-quantitative RT-PCR (Figure S3) for miR394 was consistent with the results of the quantitative RT-PCR analysis. The pathogen induced miR394 expression could be observed most clearly at 48 and 72 h post-inoculation.

**Figure 2:**
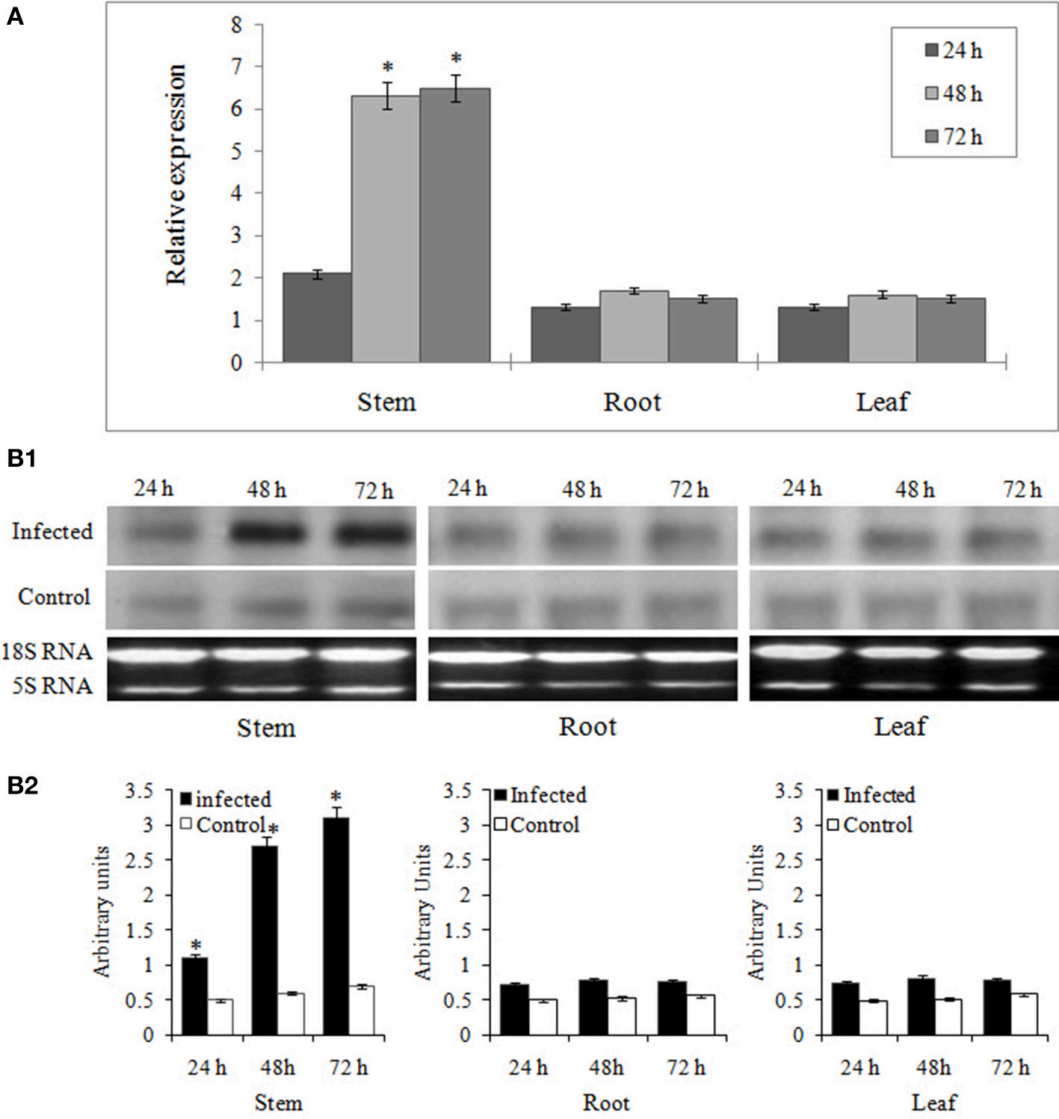
**Organ specific expression analysis of miR394 in garlic line YS4 in response to FOC**. Garlic plants were subjected to *FOC* infection. A set of five plants was collected after 0, 24, 48, and 72 h of treatment. **(A)** Analysis of miR394 expression was performed by pulsed real-time PCR (RT-PCR). Expression in the plants of 0 h and the control plants at each time was set equal to 1. The level of transcript is represented as relative expression of each miRNA with respect to its respective control. C, control; T, inoculated with FOC. The housekeeping gene was *Actin*. Error bars shows standard deviations for three independent experiments in real-time PCR. ^*^Indicates the significant difference (at *P* < 0.05) between infected and mock samples identified through two-way ANOVA test. **(B)** Expression validation of miR394 in stem, leaf and root samples by Northern blot analysis. **(B1)** Representative Northern blots and corresponding 18S rRNA from ethidium bromide stained gel **(B2)** Analysis of densitometry data as mean miRNA density/18S rRNA density ± SE. Significant difference between conditions were determined by ANOVA and Fischer's LSD test (at *P* < 0.05).

To study the organ specific induction of miR394 expression during FOC infection, we analyzed the miRNA expression profile in leaves, roots and stems of garlic cv YS4 by stem-loop RT-PCR. The results showed that miR394 was significantly induced in the set of plants treated for 48 h (6.3-fold) and 72 h (6.5-fold) in the stem tissues (Figure 2A). In contrast, the expression of miR394 was only marginally induced in both leaves and roots. In uninoculated plants, miR394 was expressed at similar levels in every tissue tested. Northern blot analysis was performed to confirm the results in the stem. miR394 expression in Northern blotting was found similar to that confirmed by stem-loop RT-PCR (Figures 2B1, B2).

### Regulation of miR394 by JA

**Figure 3:**
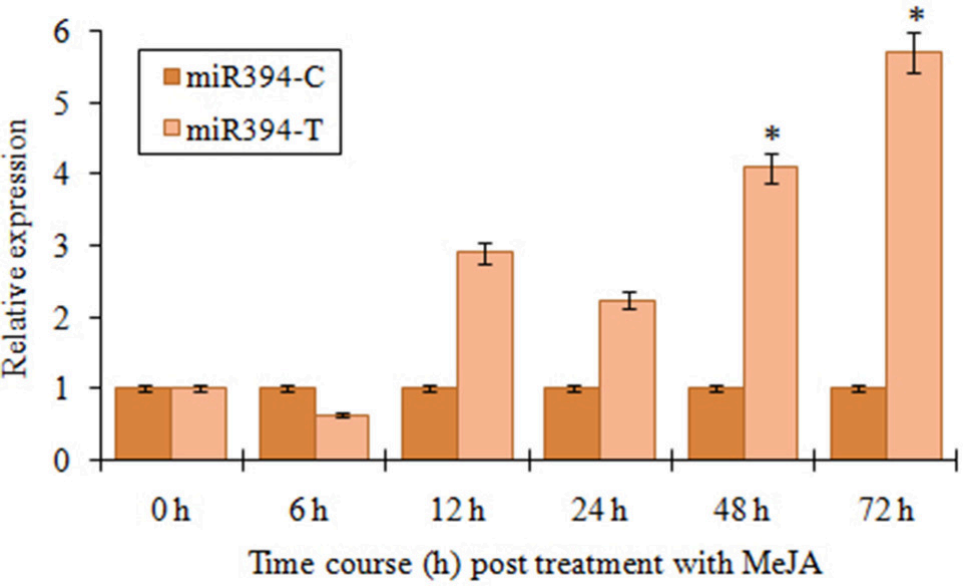
**MiR394 expression profile in response to phytohormone jasmonic acid treatment**. Garlic line YS4 grown *in vitro* was treated with MeJA. A set of five plants at each time point was collected after 0, 6, 12, 24, 48, and 72 h of treatment. The control plants were maintained in sterile water. The level of transcript is represented as relative expression of miR319 in MeJA treated plants with respect to the expression in the respective control. C, control; T, inoculated with FOC. Error bars show standard deviations for three independent experiments in real-time PCR. ^*^Indicates the significant difference at *P* < 0.05.

JA mediated signaling response is one of the most important defense mechanisms exhibited against necrotrophic phytopathogens (Antico et al., 2012). To study the JA mediated regulation of miR394, we performed the expression analysis of the miRNA in the plants treated with methyl jasmonate (MeJA) at different time points. An initial verification of JA response in garlic plants was confirmed through qRT-PCR revealing induced levels of *AsPR5* and *AsR1* genes post-treatment with MeJA (Figure S4). In garlic (YS4) plants treated with MeJA, miR394 expression decreased to half the initial levels at 6 h. At 12 h, miR394 expression increased to 2.9-fold above the control level followed by a slight decline at 24 h (2.4-fold). After that, miR394 levels gradually increased, with a maximum of 5.7-fold induction at 72 h (Figure 3). However, a second independent experiment showed a one-step response with gradual increase in miR394 expression from 12 to 72 h (Figure S5).

### Detection and validation of miR394 targets in garlic

As the plant miRNAs exhibit high homology with the cleavage site in their target mRNA, we performed a FASTA search to detect the targets of miR394 in a *de novo* assembled garlic transcriptome library. A position dependent penalty scoring system was used and targets with penalty scores of three or less were selected using the PsRNATarget server. Two potential targets were identified, an F-box protein (F-box, Contig_Asa_2307) and a Cytochrome_P450 monoxygenase protein (CYP450, Contig_Asa_3126; Table 1). Besides, another two putative targets encoding unknown and hypothetical proteins (Contig_Asa_1036; Contig_Asa_721) were also identified. Further, all the targets were predicted to be repressed through cleavage.

**Table 1:** **Prediction of miR394 targets using psRNA target**.

**Target Accn**.	**Target description**	**Expectation**	**UPE**	**miRNA aligned fragment (5′−3′)**	**Target aligned fragment (5′−3′)**	**Inhibition**
Contig_Asa_2307	F-box protein	1	11.87	UUGGCAUUCUGUCCACCUCC	GGAGGUUGACAGAAUGCCAA	Cleavage
Contig_Asa_3126	Cytochrome_P450	1	14.82	UUGGCAUUCUGUCCACCUCC	GCAGGUGGACAGAAUGCGAA	Cleavage
Contig_Asa_1036	Unknown protein	2.5	17.03	UUGGCAUUCUGUCCACCUCC	GGAGAUGGACAGGAUGCUGA	Cleavage
Contig_Asa_721	Hypothetical protein	3	18.83	UUGGCAUUCUGUCCACCUCC	GGAGGUGGAUGGAGAGCCAA	Cleavage

**Figure 4:**
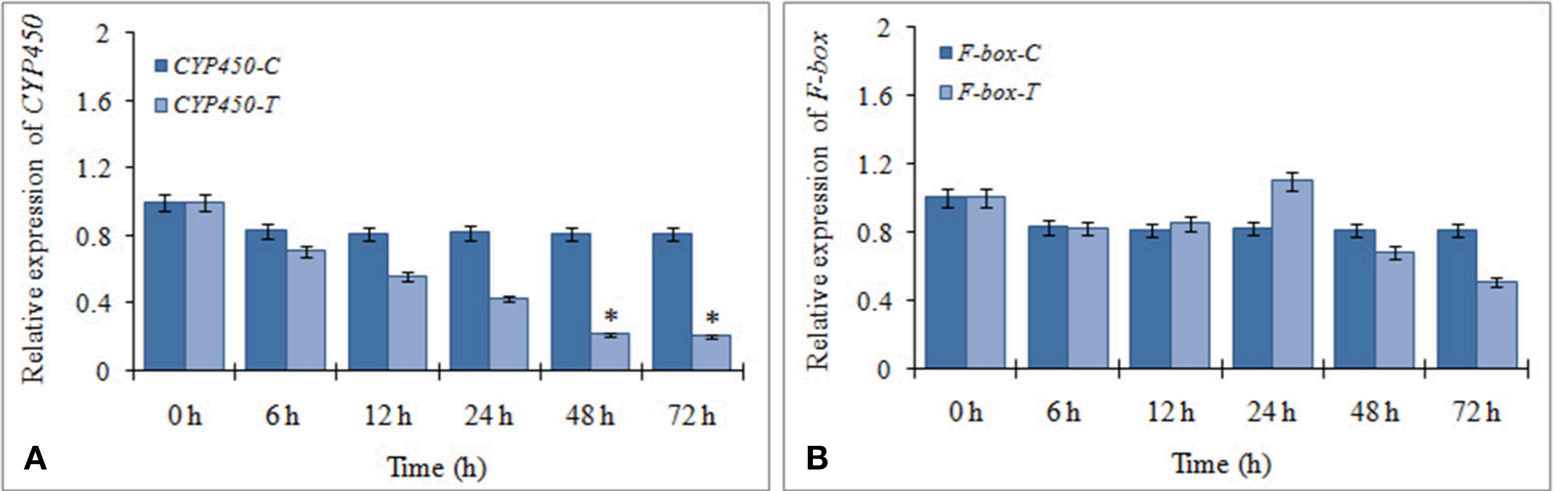
**Expression profiling of two putative miR394 targets using qRT-PCR**. **(A)** Expression of CYP450 gene post treatment with FOC, **(B)** Expression of F-box gene post treatment with FOC. Garlic line YS4 grown *in vitro* was subjected to FOC infection. *A set* of five plants at each time point was collected after 0, 6, 12, 24, 48, and 72 h of treatment. Expression in the plantlets at 0 h and in control plantlets was set equal to 1 The level of transcript is represented as relative expression of each miRNA with respect to its respective control. C, control; T, inoculated with FOC. The housekeeping gene was *Actin*. Error bars show standard deviations for three independent experiments. ^*^Indicates the significant difference (at *P* < 0.05) between infected and mock samples identified through two-way ANOVA test.

The expression profiles of the two targets (F-box and CYP450) were verified by qRT-PCR to determine their regulation during FOC infection. The results showed that CYP450 target gene was significantly down regulated in FOC inoculated garlic plants at different times and exhibited a negative relationship to the expression of miR394 (Figure 4A). In contrast, F-box gene was marginally induced by 24 h (1.1-fold) followed by gradual down regulation from 48 h (0.68-fold) to 78 h (0.51-fold) while there were no obvious changes among the corresponding expression in control group (Figure 4B). These results suggested that miR394 possibly down regulates *CYP450* gene in response to FOC infection.

**Figure 5:**
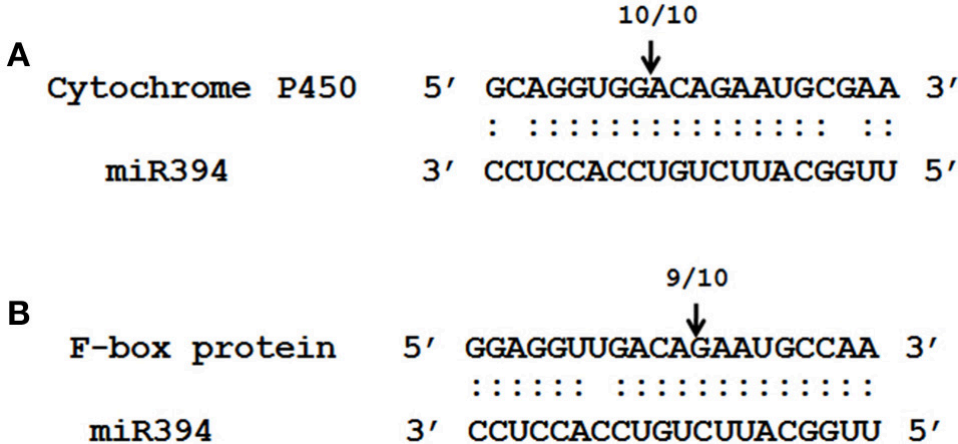
**Validation of predicted miRNA targets using RNA ligase mediated 5′ rapid amplification of cDNA ends (RACE) PCR**. Bottom strand depict the miR394 **(A,B)** while the top strand depict the target mRNA sequences from *F-box* **(A)** and *CYP450*. The arrow above sequences indicates the detected cleavage sites and the number represents independent cloned products. Matches are indicated by colon and the mismatches are unmarked.

RLM-5′ RACE analysis was performed to validate the miRNA-target interaction. RNA isolated from stem tissues at 78 h post-inoculation was used as the target genes showed maximum repression at this point. The PCR 5′ RACE products of *CYP450* and *F-box* genes were cloned and sequenced. All the ten clones sequenced from *CYP450* gene showed the cleavage site of the target after the twelfth nucleotide from the 5′ end of the miR394 (Figure 5A). Similarly, 9 out of 10 clones of *F-box* gene showed the cleavage site after the ninth nucleotide from the 5′ end of the miRNA (Figure 5B). These results indicated that both genes are direct targets of repression by miR394 directed cleavage of mRNA.

### Expression of miRNA target genes in response to JA

**Figure 6:**
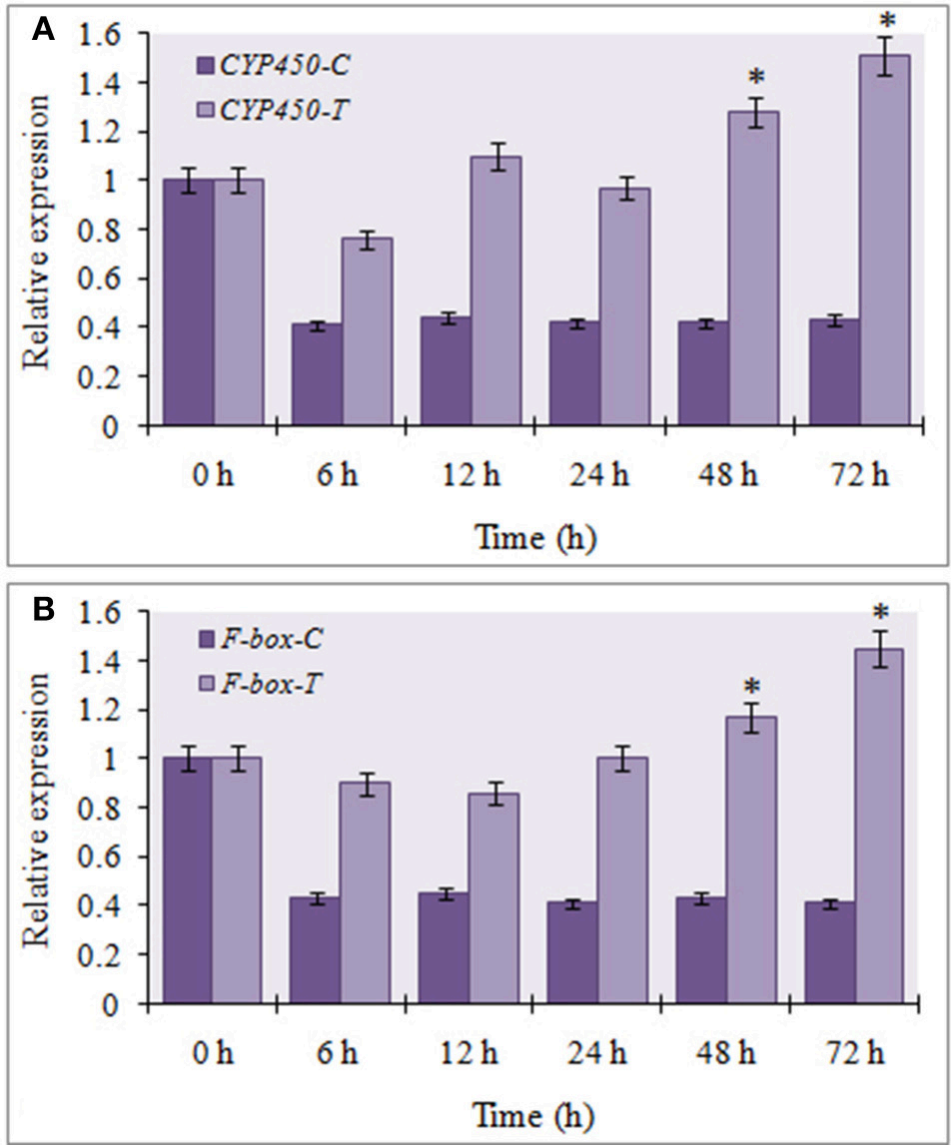
**Expression profiling of miR394 targets treated with phytohormone jasmonic acid**. **(A)** Expression of CYP450 gene post treatment with JA, **(B)** Expression of F-box gene post treatment with JA. A set of five plants was collected at each time point after 0, 6, 12, 24, 48, and 72 h of treatment. Expression in the plantlets at 0 h and in control plantlets was set equal to 1 The level of transcript is represented as relative expression of each miRNA with respect to its respective control. C, control; T, inoculated with FOC. The housekeeping gene was *Actin*. Error bars show standard deviations for three independent experiments. ^*^Indicates the significant difference at *P* < 0.05.

To determine the involvement of miR394 targets in the JA dependent defense signaling pathway, we studied the level of *CYP450* and *F-box* transcripts following temporal exogenous application of MeJA using qRT-PCR. MeJA, which is a major signaling molecule for necrotrophic pathogen response, reduced the accumulation of CYP450 transcript by 20% as early as 6 h post-treatment, which increased by 12 h (1.1-fold) followed by a slight decline at 24 h (0.99-fold). After that, CYP450 transcripts rebound upregulation at 48 h (1.28-fold) and 72 h (1.51-fold; Figure 6A). At all the time points, the transcript expressions of the treated plants were significantly higher that the respective control. The expression of F-box was reduced by 10% after 6 h of treatment before rising again to the control level after 24 h. This was followed by a gradual increase in the transcript levels of F-box from 48 h (1.17-fold) to 72 h (1.45-fold; Figure 6B). The results suggest that the expression of these target genes is enhanced by MeJA regardless of the accumulation of miR394.

### Regulation of miR394 in different garlic varieties during FOC infection

**Figure 7:**
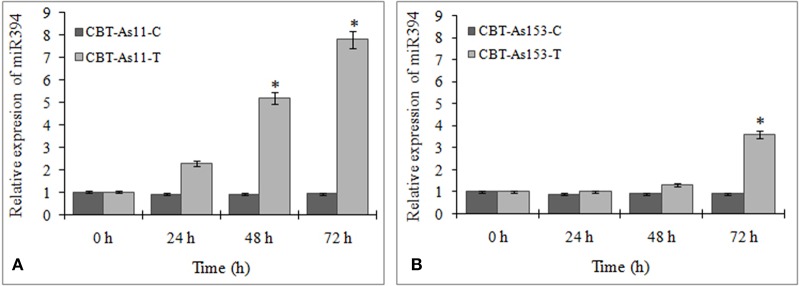
**Regulation of miR394 in genotypes of garlic with contrasting response to FOC infection**. **(A)** Relative expression of miR394 in FOC sensitive genotype CBT-As11, **(B)** Relative expression of miR394 in FOC resistant genotype CBT-As153. Garlic plants were germinated for 2 weeks and then subjected to FOC infection for 0, 24, 48, and 72 h. Expression in the plants at 0 h and in control plants was set equal to 1. The level of transcript is represented as relative expression of miR394 with respect to its respective control. C, control; T, inoculated with FOC. The housekeeping gene used was Actin. Error bars show standard deviations for three independent experiments. ^*^Indicates the significant difference (at *P* < 0.05) between infected and mock samples identified through two-way ANOVA test.

**Figure 8:**
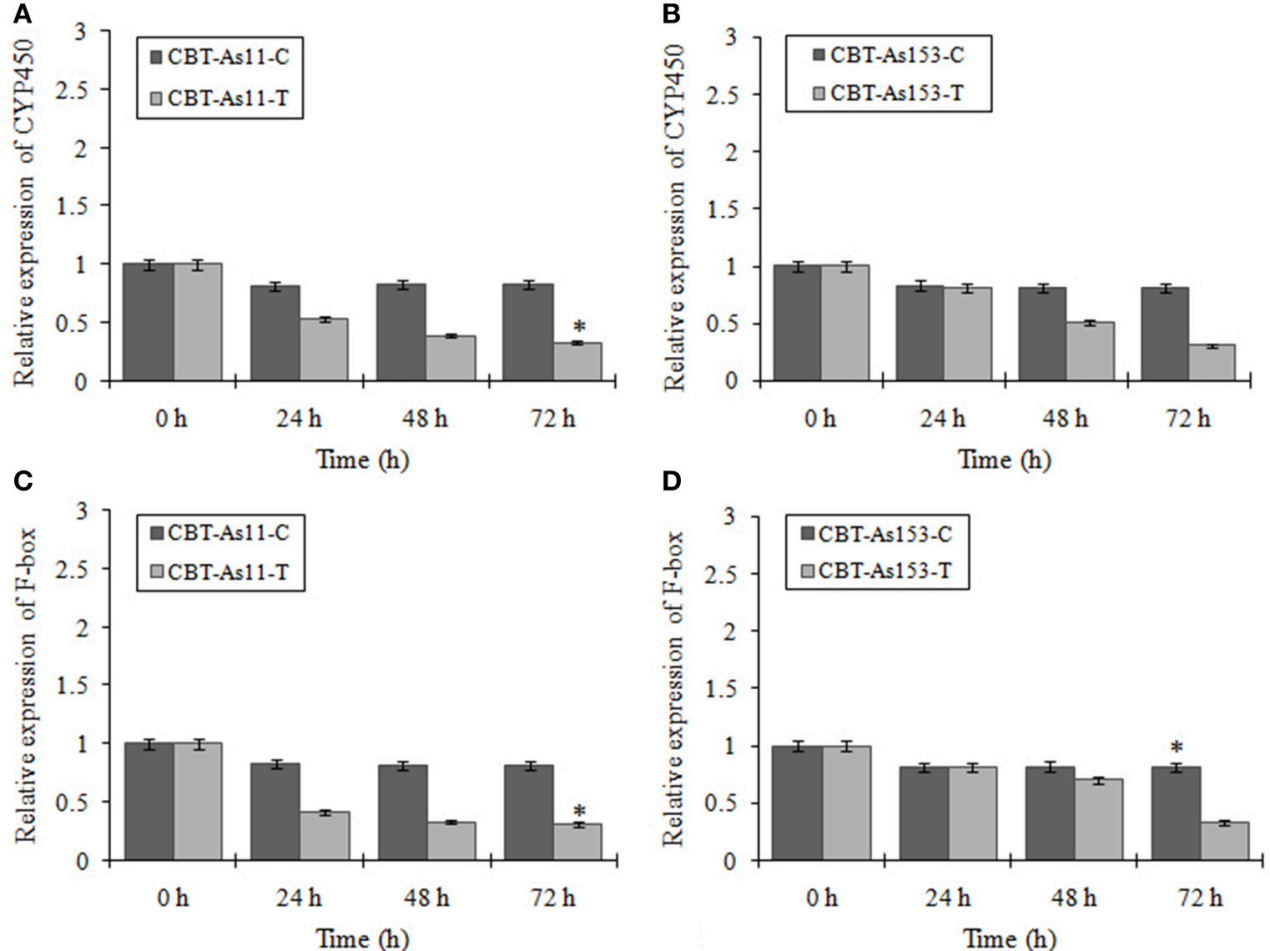
**Expression profiling of two miR394 targets (F-box and CYP450) in genotypes of garlic with contrasting response to FOC infection**. Relative expression of CYP450 gene in the sensitive genotype CBT-As11 **(A)** and resistant genotype CBT-As153 **(B)**. Relative expression of F-box gene in sensitive genotype CBT-As11 **(C)** and resistant genotype CBT-As153. Garlic plants were germinated for 2 weeks and then subjected to FOC infection for 0, 24, 48, and 72 h. Expression in the plants at 0 h and in control plants was set equal to 1. The level of transcript is represented as relative expression of gene with respect to its respective control. C, control; T, inoculated with FOC. The housekeeping gene used was Actin. Error bars show standard deviations for three independent experiments. ^*^Indicates the significant difference (at *P* < 0.05) between infected and mock samples identified through two-way ANOVA test.

To evaluate the prospect of using miR394 and its targets as markers for selection of FOC resistant garlic lines, we analyzed the regulation of miR394 and its targets in two garlic accessions CBT-As153 and CBT-As11 with variable sensitivities to FOC. The results showed that miR394 was upregulated in both the garlic lines post-inoculation with FOC for 72 h. In the FOC sensitive genotype CBT-As11, there was an early increase in the levels of miR394 after 24 h (2.3-fold), gradually increasing after 48 h (5.2-fold) and peaking at 72 h (7.8-fold; Figure 7A). On the contrary, the expression of miR394 in tolerant genotype CBT-As153 was similar to the control plants at 24 h (1.0-fold) and 48 h (1.05-fold) and significantly induced only after 72 h (3.6-fold; Figure 7B). The regulation of miR394 and its targets was also analyzed in additional eight susceptible cultivars (Bhima Omkar, Agrifound White, Yamuna Safed 8, CBT-As23, CBT-As63, CBT-As83, CBT-As103, and CBT-As171) and a wild resistant genotype (N917). The results revealed similar expression pattern of miR394 in 9 susceptible cultivars as observed previously for CBT-As11 (Figure S6). In contrast, a significant induction of miR394 was observed at 72 hpi in the resistant genotype N917 similar to that of CBT-As153 (Figure S6). This confirms the reproducible expression pattern of miR394 in response to FOC. The expression analysis of the target genes revealed that, F-box and CYP450 genes were down regulated in both the genotypes suggesting that the changes in miR394 expression was followed by inverse regulation of target mRNA (Figure 8 and Figure S7).

## Discussion

*Fusarium* basal rot caused by FOC is very difficult to control and, is responsible for severe yield losses in both standing and stored crops of onion and garlic. Although many studies have investigated the interaction of FOC with *Alliums* spp. and genes regulating FOC stress in garlic have been identified, the genetic, epigenetic and molecular mechanism of FBR resistance is still poorly understood. Recent reports have shown the involvement of miRNAs in response to fungal stresses in various plants such as *Oryza sativa* (Campo et al., 2013)*, Triticum aestivum* (Inal et al., 2014)*, Brassica napus* (Shen et al., 2014)*, Solanum lycopersicum* (Jin and Wu, 2015), and *Solanum melongena* (Yang et al., 2013). In addition, it has been revealed that pathogen induced miRNAs represses their target genes, which are significant factors involved in defense response (Ruiz-Ferrer and Voinnet, 2009). In the present study, we observed that miR394 was differentially expressed during FOC infection in a time dependent fashion (Figures 1A,B). A quick increase in miR394 following FOC infection suggest that miR394 may be involved in the defense response of garlic against FOC invasion, which encouraged us to further investigate the role of miR394 in pathogen resistance. Further, the data also showed that miR394 was significantly up-regulated in response to FOC infection in only the stem tissues and not in leaves or roots. As the basal plate or stem is the primary site of FOC infection in garlic, a strong induction of miR394 in stem might have evolved as a major determinant toward repression of target genes responsible for FBR sensitivity.

miR394 is a conserved miRNA found in several plants and has been implicated in different biological processes, ranging from drought, salinity, cold and exogenous ABA to regulation of leaf curling-related morphology in *A. thaliana* (Liu et al., 2008; Ni et al., 2012; Song et al., 2012, 2013). Additionally, miR394 has been reported to be induced in response to pathogenic fungus *Verticillium longisporum* in *B. napus* (Shen et al., 2014). Our results corroborated with a recent study in tomato in which miR394 was up-regulated in response to *Botrytis cinerea* infection (Jin and Wu, 2015). In the same study, the expression analysis revealed significant down regulation of miR394 target gene in *B. cinerea* inoculated leaves and exhibited a negative relationship to the expression of miR394 (Jin and Wu, 2015). This suggests that a transient over expression of miR394 might be playing a similar role in the regulation of FOC response in garlic. However, further functional interpretation through knockout analysis is required to confirm the regulatory role of miR394 during the interaction of garlic with FOC.

In the present study, the expression of miR394 was induced by FOC infection as well as exogenous treatment with JA (Figure 3). Molecular analysis has revealed that JA has a strong response to infection by necrotrophic fungi (Antico et al., 2012). What is more, the expressions of several defense responsive genes regulated by necrotrophic infection are significant determinants of the JA signaling pathway (Gimenez-Ibanez and Solano, 2013). Additionally, recent report has shown that the defense pathways in garlic that are activated in response to FBR are highly dependent on JA signaling (Rout et al., 2015).

Analysis of the target genes of the miRNA can aid to understand their biological roles. In this study, we identified two targets regulated by miR394 under FOC infection- Contig_Asa_2307 encoding an F-box protein and Contig_Asa_3126 encoding CYP450 protein. In *A. thaliana*, miR394 target the LCR (*Leaf Curling Responsiveness*) gene encoding an F-box protein involved in the regulation of leaf development and plant response to abiotic stresses (Ni et al., 2012; Song et al., 2012, 2013). An F-box domain protein is targeted by miR394 in *B. napus* and Lycopersicon *esculentum* in response to fungal phytopathogens (Shen et al., 2014; Jin and Wu, 2015). Furthermore, a recent report has shown that miR394 also target genes encoding flavoprotein and Cytochrome P450 during necrotrophic fungal infection (Jin and Wu, 2015).

The cleavage of the target mRNA is the principal mode of regulation by plant miRNAs (Khraiwesh et al., 2012). A 5′ RACE assay is the most useful method to detect *in vivo* product of the miRNA mediated cleaved mRNA (Jones-Rhoades et al., 2006). In the present study, 5′ RLM-RACE experiment demonstrated that miR394 is efficient in cleavage of both F-box and CYP450 genes in garlic (Figure 5). Moreover, as described previously for *B. napus* and *L. esculentum*, the cleavage of the F-box gene by miR394 occurs after the ninth nucleotide and the genes of the CYP450 family after the twelfth nucleotide from the 5′ end of the miRNA corroborated with the results obtained in the present study (Shen et al., 2014; Jin and Wu, 2015).

The transcript levels of both the targets were repressed in garlic plants exposed to FOC infection for 72 h. In addition, the regulation of FOC response mediated by miR394 was spatially specific to the stem tissue as the induction of miR394 and repression of F-box and CYP450 occur only in stems and not in leaves or roots. However, the miR394 target genes were differentially expressed in JA treated garlic plants and a direct regulation by miR394 could not be seen. While a small decrease in CYP450 and F-box mRNA levels occurred after 6 hpi, there was a gradual increase from 24 to 72 hpi for both the target genes (Figure 6A). It might be assumed that induction of the target genes by JA and their simultaneous cleavage by miR394 results in a balance reaction in the amount of the target mRNA. Similar results were also reported in sugarcane where the target genes were cleaved by miR319 under cold stress while treatment with abscisic acid (ABA) induced their expression (Thiebaut et al., 2012). Jeong et al. (2009) reported that the target mRNA levels is not always reduced completely with increase in miRNA expression. Taken together, these findings suggest that, the post-transcriptional regulation of target genes by miRNA directed cleavage might not limit the accumulation of target mRNA at all times as it could be accumulated through other defense responsive signaling pathways.

miR394 regulation was also observed in garlic varieties with contrasting response to FOC infection. Although miR394 was upregulated in both the resistant and susceptible lines post-inoculation with FOC, a temporal difference in the expression pattern was observed. While the miR394 levels increased early in susceptible line, the induction in the resistant line was observed only after 72 hpi. Similarly, the decrease in the mRNA levels of F-box and CYP450 genes was also delayed in the tolerant cultivar CBT-As153. As described in grapes and *Arabidopsis* (Narusaka et al., 2004; Cheng et al., 2010), CYP450 genes mediate the synthesis and metabolism of many primary and secondary compounds that acts as a plant defense agents against phytopathogens. Zeitlupe (ZTL) type F-box proteins have been previously implicated in defense signaling and act as positive regulator of disease resistance (Dagdas et al., 2009). Therefore, it can be suggested that miR394 mediated cleavage of its targets might act as one of the mechanism leading to susceptibility of garlic plant to FOC.

In conclusion, our data indicates that FOC induced miR394 expression possibly down regulates the target F-box and CYP450 genes, which may encode positive regulators of FOC responses. In addition, exogenous treatment of JA also regulates miR394 signaling response in garlic. An increase in the expression of genes targeted by miR394 post-treatment with JA suggest that there a balance between these two regulatory pathways in response to FOC infection. Moreover, our results also showed differences in miR394 regulation among garlic genotypes with contrasting response to FOC. The differences in the strength of regulation of miR394 and its targets could be examined as markers for selection of FOC resistant garlic cultivars.

## Author contributions

RJ conceived, designed and supervised the research work. SC performed pathogen stress treatment, RNA isolation and stem loop qPCR based expression analysis. SN performed the target identification and validation through RLM-RACE assay. SC and SN wrote the manuscript. RJ provided inputs on data presentation and critically reviewed the manuscript. All authors read and approved the final manuscript.

### Conflict of interest statement

The authors declare that the research was conducted in the absence of any commercial or financial relationships that could be construed as a potential conflict of interest.

## Supplementary material

Figure S1 **Symptoms of Fusarium basal rot on garlic. (A)** Visible difference in the growth rate of uninoculated and inoculated seedlings of the cultivar Yamuna safed 4 (YS4). **(B)** Time course (6–72 h) difference in the morphology of YS4 seedlings post-treatment with FOC. **(C)** Visible symptoms of FOC infection in garlic clove at 72 h post-inoculation (hpi). **(D)** Development of basal rot symptoms in the storage bulbs by 72 hpi. C- Uninoculated control, T- Inoculated with FOC, hpi- hours post-inoculation.

Figure S2 **Validation of the FOC treatment through analysis of FOC responsive genes AsPR5 and AsR1**. Garlic plants (YS4) grown *in vitro* were treated with FOC. A set of five plants was collected at each point after 0, 6, 12, 24, 48, and 72 h of treatment. Each point of the experiment had its own control. At 0 h the relative expression is equal to 1. The level of transcript is represented as a relative expression of the markers of stress (AsPR5 and AsR1) with respect to the corresponding control. The housekeeping gene was *Actin*. Error bars show standard deviations for three independent experiments in real-time PCR. ^*^Indicates significant difference at *P* < 0.05.

Figure S3 **Temporal expression analysis of garlic miR394 in response to FOC using semi-quantitative reverse transcription PCR**. The housekeeping gene used as control was garlic *Actin*.

Figure S4 **Expression of FOC responsive genes AsPR5 and AsR1 post-treatment with MeJA**. Garlic plants (YS4) grown *in vitro* were treated with MeJA. A set of five plants was collected at each point after 0, 6, 12, 24, 48, and 72 h of treatment. Each point of the experiment had its own control. At 0 h the relative expression is equal to 1. The level of transcript is represented as a relative expression of the markers of stress (AsPR5 and AsR1) with respect to the corresponding control. C, control; T, inoculated with FOC. The housekeeping gene was *Actin*. Error bars show standard deviations for three independent experiments in real-time PCR. ^*^Indicates significant difference at *P* < 0.05.

Figure S5 **MiR394 expression profile in a second JA treatment**. Garlic line YS4 grown *in vitro* were treated with MeJA. A set of three plants at each time point was collected after 0, 6, 12, 24, 48, and 72 h of treatment. The control plants were maintained in sterile water. The level of transcript is represented as relative expression of miR319 in MeJA treated plants with respect to the expression in the respective control. C, control; T, inoculated with FOC. The housekeeping gene was Actin. Error bars show standard deviations for three independent experiments in real-time PCR.

Figure S6 **Regulation of miR394 in additional genotypes of garlic with contrasting response to FOC infection. N917- Resistant; Bhima Omkar (BO), Agrifound White (AW), Yamuna Safed 8 (YS8), CBT-As23, CBT-As63, CBT-As83, CBT_As103 and CBT-As171- sensitive**. Garlic plants were germinated for 2 weeks and then subjected to FOC infection for 0, 24, 48, and 72 h. Expression in the plants at 0 h and in control plants was set equal to 1. The level of transcript is represented as relative expression of miR394 with respect to its respective control. C, control; T, inoculated with FOC. The housekeeping gene used was *Actin*. C, control; T, inoculated with FOC. Error bars show standard deviations for three independent experiments. ^*^Indicates the significant difference (at *P* < 0.05) between infected and mock samples identified through two-way ANOVA test.

Figure S7 **Expression profiling of miR394 target (CYP450) in additional genotypes of garlic with contrasting response to FOC infection. N917- Resistant; Bhima Omkar (BO), Agrifound White (AW), Yamuna Safed 8 (YS8), CBT-As23, CBT-As63, CBT-As83, CBT_As103 and CBT-As171- sensitive**. Garlic plants were germinated for 2 weeks and then subjected to FOC infection for 0, 24, 48 and 72 h. Expression in the plants at 0 h and in control plants was set equal to 1. The level of transcript is represented as relative expression of gene with respect to its respective control. C, control; T, inoculated with FOC. The housekeeping gene used was Actin. Error bars show standard deviations for three independent experiments. ^*^Indicates the significant difference (at *P* < 0.05) between infected and mock samples identified through two-way ANOVA test.

Table S1 **List of miRNAs used in the present study**.

Table S2 **Sequences of oligonucleotides used for stem-loop qRT-PCR**.

Table S3 **Primers sequences used for qPCR amplification of putative targets of miR394**.

Table S4 **Primers used in 5′ RLM-RACE assay**.
