# Supplementary figures and images for: Regulation of miR394 in Response to Fusarium oxysporum f. sp. cepae (FOC) Infection in Garlic (Allium sativum L)
Source: Front Plant Sci. 2016 Mar 4;7:258. doi: 10.3389/fpls.2016.00258 (PMC4777725; doi:10.3389/fpls.2016.00258)

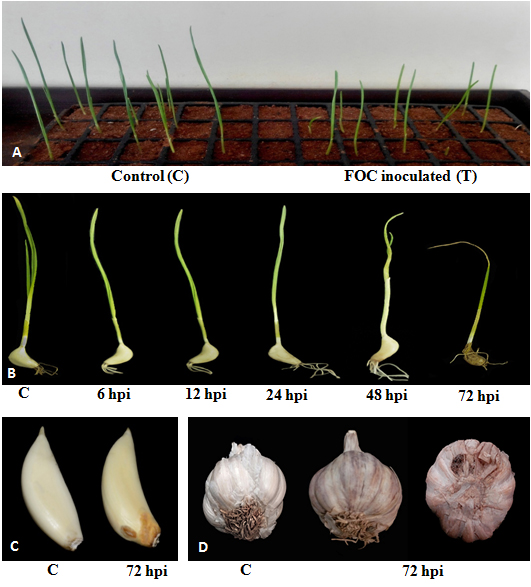

Supplement: Figure S1 — Symptoms of Fusarium basal rot on garlic. (A) Visible difference in the growth rate of uninoculated and inoculated seedlings of the cultivar Yamuna safed 4 (YS4). (B) Time course (6–72 h) difference in the morphology of YS4 seedlings post-treatment with FOC. (C) Visible symptoms of FOC infection in garlic clove at 72 h post-inoculation (hpi). (D) Development of basal rot symptoms in the storage bulbs by 72 hpi. C- Uninoculated control, T- Inoculated with FOC, hpi- hours post-inoculation. [file Image1.jpg]

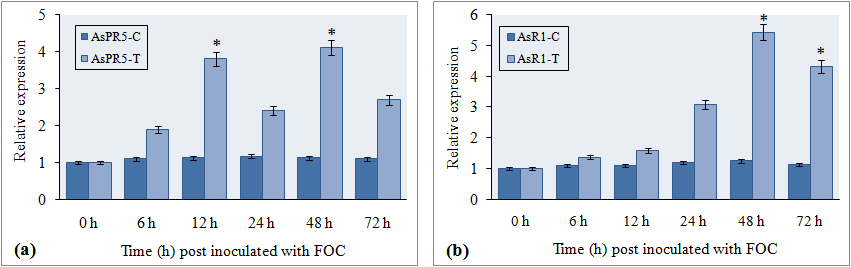

Supplement: Figure S2 — Validation of the FOC treatment through analysis of FOC responsive genes AsPR5 and AsR1. Garlic plants (YS4) grown in vitro were treated with FOC. A set of five plants was collected at each point after 0, 6, 12, 24, 48, and 72 h of treatment. Each point of the experiment had its own control. At 0 h the relative expression is equal to 1. The level of transcript is represented as a relative expression of the markers of stress (AsPR5 and AsR1) with respect to the corresponding control. The housekeeping gene was Actin. Error bars show standard deviations for three independent experiments in real-time PCR. *Indicates significant difference at P < 0.05. [file Image2.JPEG]

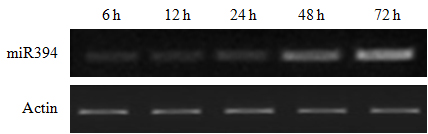

Supplement: Figure S3 — Temporal expression analysis of garlic miR394 in response to FOC using semi-quantitative reverse transcription PCR. The housekeeping gene used as control was garlic Actin. [file Image3.JPEG]

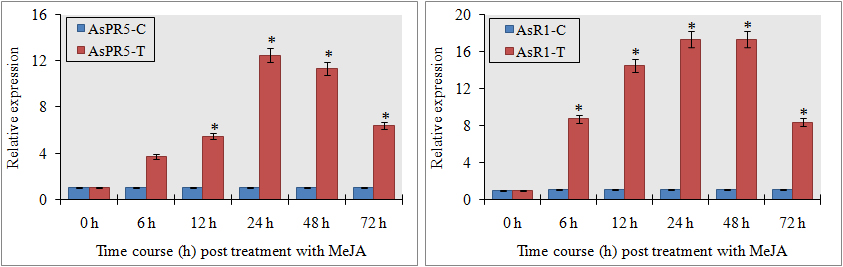

Supplement: Figure S4 — Expression of FOC responsive genes AsPR5 and AsR1 post-treatment with MeJA. Garlic plants (YS4) grown in vitro were treated with MeJA. A set of five plants was collected at each point after 0, 6, 12, 24, 48, and 72 h of treatment. Each point of the experiment had its own control. At 0 h the relative expression is equal to 1. The level of transcript is represented as a relative expression of the markers of stress (AsPR5 and AsR1) with respect to the corresponding control. C, control; T, inoculated with FOC. The housekeeping gene was Actin. Error bars show standard deviations for three independent experiments in real-time PCR. *Indicates significant difference at P < 0.05. [file Image4.JPEG]

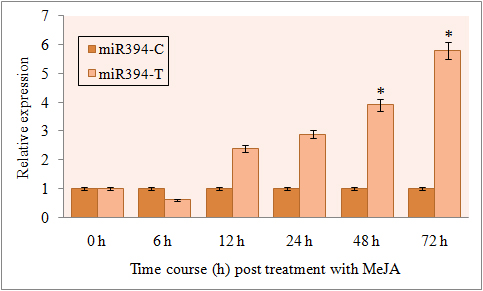

Supplement: Figure S5 — MiR394 expression profile in a second JA treatment. Garlic line YS4 grown in vitro were treated with MeJA. A set of three plants at each time point was collected after 0, 6, 12, 24, 48, and 72 h of treatment. The control plants were maintained in sterile water. The level of transcript is represented as relative expression of miR319 in MeJA treated plants with respect to the expression in the respective control. C, control; T, inoculated with FOC. The housekeeping gene was Actin. Error bars show standard deviations for three independent experiments in real-time PCR. [file Image5.JPEG]

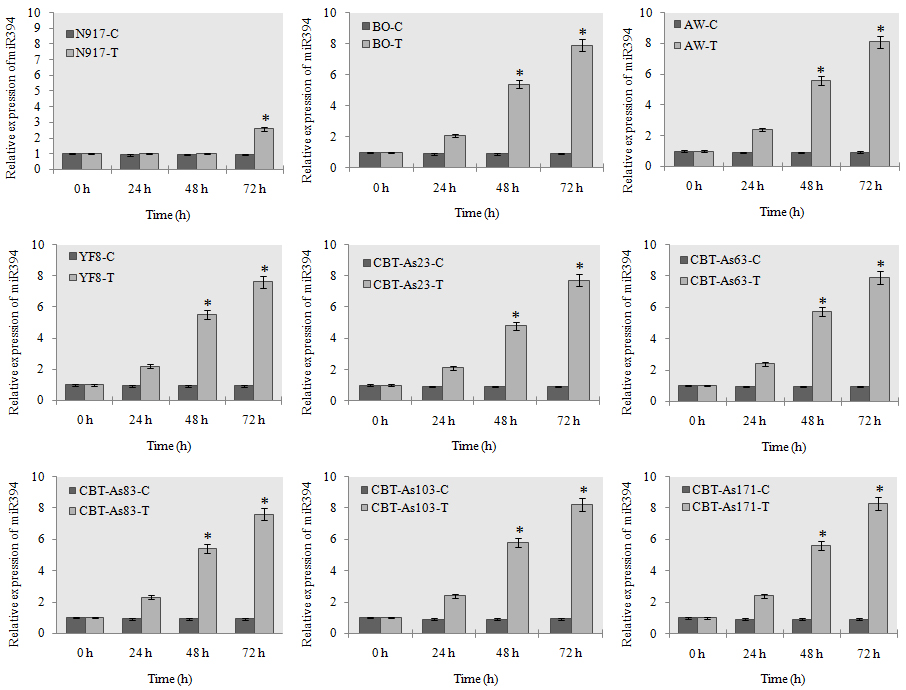

Supplement: Figure S6 — Regulation of miR394 in additional genotypes of garlic with contrasting response to FOC infection. N917- Resistant; Bhima Omkar (BO), Agrifound White (AW), Yamuna Safed 8 (YS8), CBT-As23, CBT-As63, CBT-As83, CBT_As103 and CBT-As171- sensitive. Garlic plants were germinated for 2 weeks and then subjected to FOC infection for 0, 24, 48, and 72 h. Expression in the plants at 0 h and in control plants was set equal to 1. The level of transcript is represented as relative expression of miR394 with respect to its respective control. C, control; T, inoculated with FOC. The housekeeping gene used was Actin. C, control; T, inoculated with FOC. Error bars show standard deviations for three independent experiments. *Indicates the significant difference (at P < 0.05) between infected and mock samples identified through two-way ANOVA test. [file Image6.JPEG]

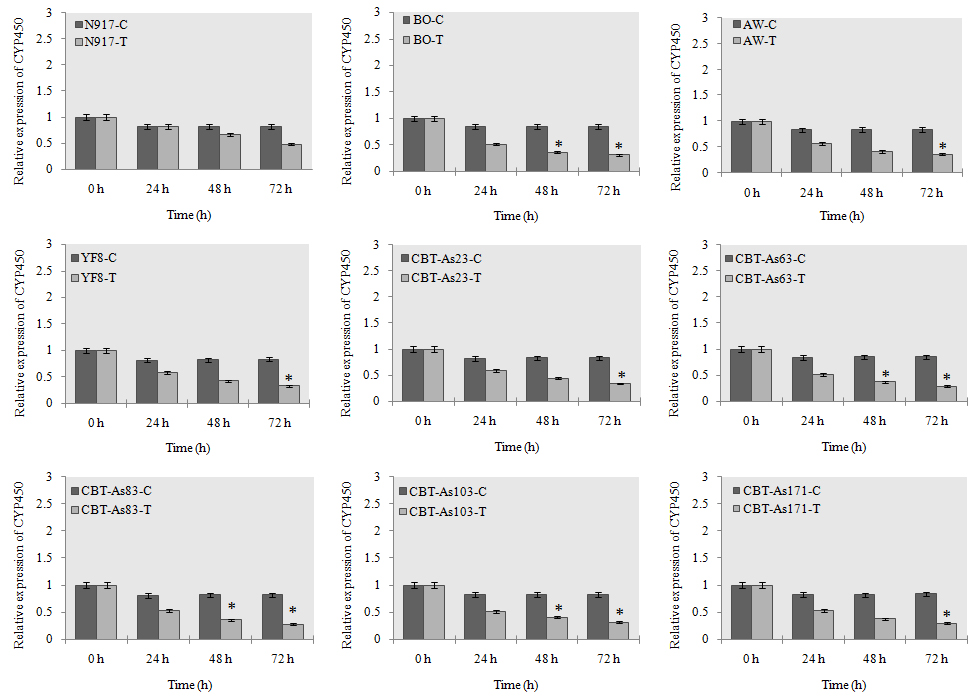

Supplement: Figure S7 — Expression profiling of miR394 target (CYP450) in additional genotypes of garlic with contrasting response to FOC infection. N917- Resistant; Bhima Omkar (BO), Agrifound White (AW), Yamuna Safed 8 (YS8), CBT-As23, CBT-As63, CBT-As83, CBT_As103 and CBT-As171- sensitive. Garlic plants were germinated for 2 weeks and then subjected to FOC infection for 0, 24, 48 and 72 h. Expression in the plants at 0 h and in control plants was set equal to 1. The level of transcript is represented as relative expression of gene with respect to its respective control. C, control; T, inoculated with FOC. The housekeeping gene used was Actin. Error bars show standard deviations for three independent experiments. *Indicates the significant difference (at P < 0.05) between infected and mock samples identified through two-way ANOVA test. [file Image7.JPEG]
