# Supplementary material for: Regulation of miR394 in Response to Fusarium oxysporum f. sp. cepae (FOC) Infection in Garlic (Allium sativum L)
Source: Front Plant Sci. 2016 Mar 4;7:258. doi: 10.3389/fpls.2016.00258 (PMC4777725; doi:10.3389/fpls.2016.00258)
Supplement: Table S1 — List of miRNAs used in the present study. [file Table1.DOCX]

**Table S1: List of miRNAs used in the present study**

| Sl no. | miRNA | Sequence (5’- 3’) |
| --- | --- | --- |
| 1 | miR156 | UUGACAGAAGAAAGAGAGCA |
| 2 | miR159 | UUUGGAUUGAAGGGAGCUCU |
| 3 | miR169 | UGAGCCAAAGAUGACUUGCC |
| 4 | miR319 | UUGGACUGAAGGGAGCUCCC |
| 5 | miR394 | UUGGCAUUCUGUCCACCUCC |
| 6 | miR482 | UCUUCCUUGUUCCUCCCAUU |
