# Supplementary material for: Regulation of miR394 in Response to Fusarium oxysporum f. sp. cepae (FOC) Infection in Garlic (Allium sativum L)
Source: Front Plant Sci. 2016 Mar 4;7:258. doi: 10.3389/fpls.2016.00258 (PMC4777725; doi:10.3389/fpls.2016.00258)
Supplement: Table S2 — Sequences of oligonucleotides used for stem-loop qRT-PCR. [file Table2.DOCX]

**Table S2: Sequences of oligonucleotides used for stem-loop qRT-PCR.**

| **miRNAs** | **Stem-loop RT primer** | **miRNA specific forward primer** |
| --- | --- | --- |
| miR156 | GTCGGCTCTAGTGCAGGGTCCGAGGTATTCGCACCGGATACGACTGCTCT | GGCGCGTTGACAGAAGAAGA |
| miR159 | GTCGGCTCTAGTGCAGGGTCCGAGGTATTCGCACCGGATACGACAGAGCT | GGCTTTGGATTGAAGGGAGCT |
| miR169 | GTCGGCTCTAGTGCAGGGTCCGAGGTATTCGCACCGGATACGACGGCAAG | GGCGCGTGAGCCAAAGATGAG |
| miR319 | GTCGGCTCTAGTGCAGGGTCCGAGGTATTCGCACCGGATACGACGGGAGC | GCGTTGGACTGAAGGGAGCT |
| miR394 | GTCGGCTCTAGTGCAGGGTCCGAGGTATTCGCACCGGATACGACGGAGGT | GGCTTGGCATTCTGTCCACCT |
| miR482 | GTCGGCTCTAGTGCAGGGTCCGAGGTATTCGCACCGGATACGACTAGGAA | GCGTCTTCCTTGTTCCTCCCA |

Universal reverse primer- GTGCAGGGTCCGAGGT
