# Supplementary material for: Regulation of miR394 in Response to Fusarium oxysporum f. sp. cepae (FOC) Infection in Garlic (Allium sativum L)
Source: Front Plant Sci. 2016 Mar 4;7:258. doi: 10.3389/fpls.2016.00258 (PMC4777725; doi:10.3389/fpls.2016.00258)
Supplement: Table S3 — Primers sequences used for qPCR amplification of putative targets of miR394. [file Table3.DOCX]

**Table S3: Primers sequences used for qPCR amplification of putative targets of miR394**

| **Sl no.** | **Targets** |  | **Sequence (5’-3’)** |
| --- | --- | --- | --- |
| 1 | CYP450 | Forward | GCGTGCAGTCCACCAAGAT |
|  |  | Reverse | GAGGTTGTACATGTAGCACGAGATG |
| 2 | F-box | Forward | GCCTCCTGTGCCTTTTGAGA |
|  |  | Reverse | CCGGCATATCCCAGAACACT |
