# Supplementary material for: Regulation of miR394 in Response to Fusarium oxysporum f. sp. cepae (FOC) Infection in Garlic (Allium sativum L)
Source: Front Plant Sci. 2016 Mar 4;7:258. doi: 10.3389/fpls.2016.00258 (PMC4777725; doi:10.3389/fpls.2016.00258)
Supplement: Table S4 — Primers used in 5′ RLM-RACE assay. [file Table4.DOCX]

**Table S4: Primers used in 5’RLM-RACE assay**

| **Sl no.** | **Primer** | **Sequence (5’-3’)** |
| --- | --- | --- |
| 1 | GeneRacer™ 5′ Primer | CGACTGGAGCACGAGGACACTGA |
| 2 | GeneRacer™ 5′ Nested Primer | GGACACTGACATGGACTGAAGGAGTA |
| 3 | Gene specific primer F-box 1R | GGAATAGACACAGGCATAGACACG |
| 4 | Gene specific primer F-box 2R (nested) | CAGGCATAGACACGATTATTAGCAC |
| 5 | Gene specific primer CYP450-1R | CCATGGTCAATCTAGGGTCTCTAC |
| 6 | Gene specific primer CYP450-2R (nested) | TCAATCTAGGGTCTCTACGTGTGAT |
